# Supplementary material for: Ligand-Length Modification in CsPbBr3 Perovskite Nanocrystals and Bilayers with PbS Quantum Dots for Improved Photodetection Performance
Source: Nanomaterials (Basel). 2020 Jul 2;10(7):1297. doi: 10.3390/nano10071297 (PMC7408175; doi:10.3390/nano10071297)
Supplement: Supplementary file 1 [file nanomaterials-10-01297-s001.pdf]

nanomaterials

MDPI

Ligand-Length Modification in $CsPbBr_3$ Perovskite Nanocrystals and Bilayers with PbS Quantum Dots for Improved Photodetection Performance

Supplementary Information

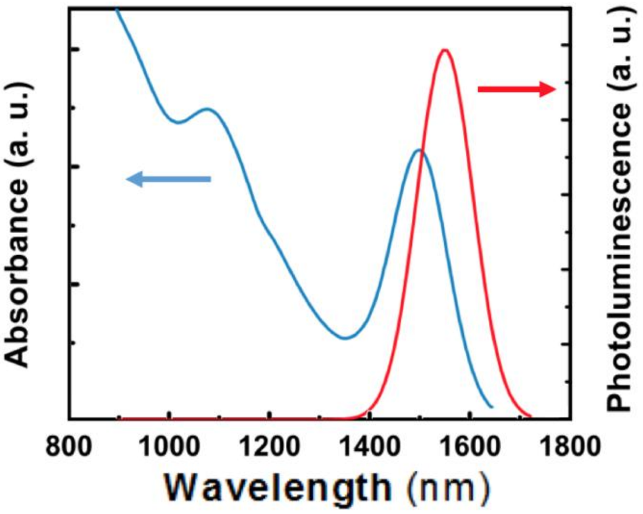

Figure S1. PL (red curve) and absorbance (blue curve) of colloidal PbS QDs used in this work for preparation of films in tandem devices with films of $CsPbBr_3$ PNCs.

In order to confirm the ligand exchange, the corresponding FTIR spectra of the $CsPbBr_3$ NCs thin films were measured before and after the ligand exchange as shown in Figure S2. OA-OAm-capped $CsPbBr_3$ films show two intense peaks at 2854 cm^{-1} and 2924 cm^{-1} that can be assigned to C–H stretching of methylene (–$(CH_2)_n$–) in long alkyl chain of OA and OAm. Similarly, the peaks at 1464 and 722 cm^{-1} are also ascribe to C–H bending of long alkyl chain. The broad peak observed at 3460 cm^{-1} indicates the presence of O–H and N–H stretching of OA and OAm. The peak at 1735 cm^{-1} is characteristic of C=O stretching of carboxylic acid. On the other hand, the solid-state ligand exchange of OA and OAm by MPA is confirmed by the strong decrease in intensities of the aliphatic C–H stretching peaks at 2918 and 2845cm^{-1} of methylene (–$(CH_2)_n$–) in long alkyl chain of OA and OAm. The very broad peak observed at 3460 cm^{-1} indicates the presence of internally bonded OH stretching (from H bonding between carboxylic acid of MPA). In addition, the peaks at 1627 cm−1 can be assigned to the vibration of the carboxylate anions of 3-MPA molecules coordinated to Pb(II) of $CsPbBr_3$ [1].

The absence of C-S and C-S-H stretching vibrations is expected since they normally give rise to very weak absorptions in the infrared spectrum. However the peak at 750 cm^{-1} can be attributed to C-S stretching of disulfides (S-S) [2].

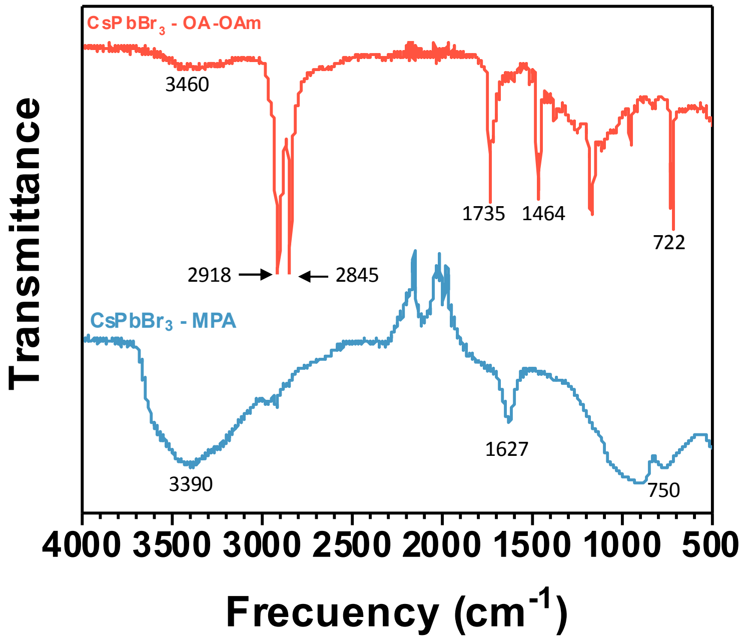

Figure S2. FTIR spectra measured (with the ATR Agilent Cary 630 setup) in a film of a pristine film of OA-OAm-capped $CsPbBr_3$ PNCs (red continuous line) and the same film after MPA ligand exchange procedure (blue continuous line).

In the band diagram shown in Figure S3 (energy levels extracted from references [3–5]) it can be observed how the charge separation is provided by the structure. As in other devices, the MoO_3 oxide interlayer plays the role of high-energy electron blocking layer [6], while allowing hole transfer towards the gold electrode.

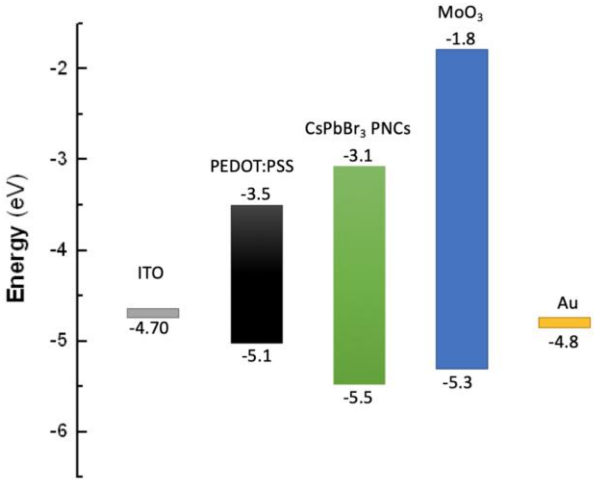

Figure S3. Schematic energy level diagram of a complete photovoltaic device based on the light absorbing layer of $CsPbBr_3$ PNCs.

Table S1. Comparison of perovskite-based photodetectors.

Type	Architecture	Material	R (A/W)	D^* (jones)	Rise/Decay (ms)	Year	Ref
Photoconductor	ITO/$CsPbCl_3$/ITO	0D	1.89	-	41/43	2017	[7]
MSM	Au/$CsPbBr_3$/Au	Thin films	55	-	0.43/0.318	2017	[8]
MSM	Au/$CsPbBr_3$ – TiO_2 /Au	0D	3.5	-	> 1000	2017	[9]
MSM	Au/$CsPbBr_3$ /Au	2D	1.33	0.86×10^{12}	20.9/24.6	2018	[10]
Phototransistor	$CsPbBr_3$/MoS_2	0D/2D	4.4	2.5×10^{10}	0.72/1.01	2018	[11]
Phototransistor	$CsPbBr_3$/MoS_2	0D/2D	4×10^4	-	7.5/8	2019	[12]
Photodiode	ITO/$CH_3NH_3PbI_3$/Au	2D	0.036	-	320/330	2017	[13]
Photodiode	FTO/TiO_2/$CsPbBr_3$/Spiro-OMeTAD/Au	0D	3	1×10^{14}	-	2018	[14]
Photodiode	ITO/PEDOT:PSS/$CsPbBr_3$/MoO_3/Au	0D	0.1	8×10^{10}	2/1.5	2019	Here

References

1. Catalano, J.; Murphy, A.; Yao, Y.; Yap, G.P.A.; Zumbulyadis, N.; Centeno, S.A.; Dybowski, C. Coordination geometry of lead carboxylates – spectroscopic and crystallographic evidence. *Dalt. Trans.* **2015**, *44*, 2340–2347.
2. Coates, J. Interpretation of Infrared Spectra, A Practical Approach. *Encycl. Anal. Chem.* **2006**.
3. Hori, T.; Moritou, H.; Fukuoka, N.; Sakamoto, J.; Fujii, A.; Ozaki, M. Photovoltaic Properties in Interpenetrating Heterojunction Organic Solar Cells Utilizing MoO3 and ZnO Charge Transport Buffer Layers. *Materials (Basel).* **2010**, *3*, 4915–4921.
4. Golubev, T.; Liu, D.; Lunt, R.; Duxbury, P. Understanding the impact of C60 at the interface of perovskite solar cells via drift-diffusion modeling. *AIP Adv.* **2019**, *9*, 35026.
5. Moyen, E.; Kanwat, A.; Cho, S.; Jun, H.; Aad, R.; Jang, J. Ligand removal and photo-activation of CsPbBr3 quantum dots for enhanced optoelectronic devices. *Nanoscale* **2018**, *10*, 8591–8599.
6. Ng, C.H.; Ripolles, T.S.; Hamada, K.; Teo, S.H.; Lim, H.N.; Bisquert, J.; Hayase, S. Tunable Open Circuit Voltage by Engineering Inorganic Cesium Lead Bromide/Iodide Perovskite Solar Cells. *Sci. Rep.* **2018**, *8*, 2482.
7. Zhang, J.; Wang, Q.; Zhang, X.; Jiang, J.; Gao, Z.; Jin, Z.; Liu, S. (Frank) High-performance transparent ultraviolet photodetectors based on inorganic perovskite $CsPbCl_3$ nanocrystals. *RSC Adv.* **2017**, *7*, 36722–36727.
8. Li, Y.; Shi, Z.F.; Li, S.; Lei, L.Z.; Ji, H.F.; Wu, D.; Xu, T.T.; Tian, Y.T.; Li, X.J. High-performance perovskite photodetectors based on solution-processed all-inorganic CsPbBr3 thin films. *J. Mater. Chem. C* **2017**, *5*, 8355–8360.
9. Zhou, L.; Yu, K.; Yang, F.; Zheng, J.; Zuo, Y.; Li, C.; Cheng, B.; Wang, Q. All-inorganic perovskite quantum dot/mesoporous TiO2 composite-based photodetectors with enhanced performance. *Dalt. Trans.* **2017**, *46*, 1766–1769.
10. Li, Y.; Shi, Z.; Lei, L.; Zhang, F.; Ma, Z.; Wu, D.; Xu, T.; Tian, Y.; Zhang, Y.; Du, G.; et al. Highly Stable Perovskite Photodetector Based on Vapor-Processed Micrometer-Scale $CsPbBr_3$ Microplatelets. *Chem. Mater.* **2018**, *30*, 6744–6755.
11. Song, X.; Liu, X.; Yu, D.; Huo, C.; Ji, J.; Li, X.; Zhang, S.; Zou, Y.; Zhu, G.; Wang, Y.; et al. Boosting Two-Dimensional MoS_2 /$CsPbBr_3$ Photodetectors via Enhanced Light Absorbance and Interfacial Carrier Separation. *ACS Appl. Mater. Interfaces* **2018**, *10*, 2801–2809.
12. Lin, R.; Li, X.; Zheng, W.; Huang, F. Balanced Photodetection in Mixed-Dimensional Phototransistors Consisting of $CsPbBr_3$ Quantum Dots and Few-Layer MoS_2. *ACS Appl. Nano Mater.* **2019**, *2*, 2599–2605.

13. Li, P.; Shivananju, B.N.; Zhang, Y.; Li, S.; Bao, Q. High performance photodetector based on 2D $CH_3NH_3PbI_3$ perovskite nanosheets. *J. Phys. D. Appl. Phys.* **2017**, *50*, 094002.
14. Yang, Z.; Wang, M.; Li, J.; Dou, J.; Qiu, H.; Shao, J. Spray-Coated $CsPbBr_3$ Quantum Dot Films for Perovskite Photodiodes. *ACS Appl. Mater. Interfaces* **2018**, *10*, 26387–26395.
